# The value of the malignant subregion-based texture analysis in predicting the Ki-67 status in breast cancer

**DOI:** 10.3389/fonc.2024.1359925

**Published:** 2024-05-21

**Authors:** Chao Hua, Chen Wenwen, Rui Huijuan, Pan Ting, Zhang Jin

**Affiliations:** Department of Radiology, Changzhou Cancer Hospital, Changzhou, China

**Keywords:** breast cancer, texture analysis, malignant subregion, Ki-67 expression, machine learning

## Abstract

**Objective:**

To evaluate the value of the malignant subregion-based texture analysis in predicting Ki-67 status in breast cancer.

**Materials and methods:**

The dynamic contrast-enhanced magnetic resonance imaging data of 119 histopathologically confirmed breast cancer patients (81 patients with high Ki-67 expression status) from January 2018 to February 2023 in our hospital were retrospectively collected. According to the enhancement curve of each voxel within the tumor, three subregions were divided: washout subregion, plateau subregion, and persistent subregion. The washout subregion and the plateau subregion were merged as the malignant subregion. The texture features of the malignant subregion were extracted using Pyradiomics software for texture analysis. The differences in texture features were compared between the low and high Ki-67 expression cohorts and then the receiver operating characteristic (ROC) curve analysis to evaluate the predictive performance of texture features on Ki-67 expression. Finally, a support vector machine (SVM) classifier was constructed based on differential features to predict the expression level of Ki-67, the performance of the classifier was evaluated using ROC analysis and confirmed using 10-fold cross-validation.

**Results:**

Through comparative analysis, 51 features exhibited significant differences between the low and high Ki-67 expression cohorts. Following feature reduction, 5 features were selected to build the SVM classifier, which achieved an area under the ROC curve (AUC) of 0.77 (0.68–0.87) for predicting the Ki-67 expression status. The accuracy, sensitivity, and specificity were 0.76, 0.80, and 0.68, respectively. The average AUC from the 10-fold cross-validation was 0.72 ± 0.14.

**Conclusion:**

The texture features of the malignant subregion in breast cancer were potential biomarkers for predicting Ki-67 expression level in breast cancer, which might be used to precisely diagnose and guide the treatment of breast cancer.

## Introduction

According to global cancer statistics in 2020, female breast cancer has surpassed lung cancer as the most commonly diagnosed cancer, and the leading cause of cancer death in women (1). With the advancement of cancer diagnosis and treatment technologies, the prognosis of breast cancer in developing countries has significantly improved, yet there remains a substantial gap compared to developed countries.

Ki-67, a marker of cellular proliferation, plays a crucial role in indicating the malignancy and prognosis of breast tumors, and high Ki-67 expression conferred a worse prognosis (2). Therefore, accurate assessment of Ki-67 expression level is instrumental in the diagnosis and treatment of breast cancer (3). Dynamic contrast-enhanced magnetic resonance imaging (DCE-MRI) of the breast is now widely used in routine examinations for breast cancer, and high-risk women screening with DCE-MRI is more effective than either mammography and/or ultrasound (4). The time-intensity curve (TIC) of contrast enhancement in breast tumors is also indicative of the malignancy level of breast tumors. Considering the heterogeneity within tumors (5), different voxels in a lesion exhibit varied enhancement TICs, among which the malignant subregion, composed of voxels with a specific enhancement pattern, is closely related to the malignancy (6, 7). However, few of studies have investigated the association between the characteristics of the malignant subregion with the Ki-67 status.

Texture analysis is an advanced method in medical image analysis that converts images into high-throughput texture features. By applying statistical analysis or machine learning techniques, it can identify the potential imaging biomarkers to assist clinical diagnosis and treatment. Texture analysis has been widely used in breast cancer study, and have been proven a promising way to achieve precise medicine (8–11). The high-throughput texture features could identify the invisible information, which might be associated with pathological and molecular phenotype information (12).

This study aimed to extract texture features from the malignant subregion of breast lesions and assessed their ability to predict Ki-67 expression status.

## Materials and methods

### Patients

This study was approved by the Research Ethics Committees of our hospitals, with the need for informed consent waived for this retrospective study. The study included 119 patients who underwent preoperative breast DCE-MR examinations at our hospital from January 2018 to February 2023. All patients were pathologically confirmed as breast cancer. All patients were female, with an average age of 54.19 ± 11.05 years. Inclusion criteria: (1) All patients underwent breast MR examinations before surgery; (2) All patients underwent surgical treatment and specimen immunohistochemical analysis. Exclusion criteria: (1) Concurrent other malignant tumors; (2) Treatment related to breast cancer, such as radiotherapy or chemotherapy, before the MR scan. According to the St Gallen International Expert Consensus (13), Ki-67, a marker of cellular proliferation, plays a crucial role in indicating the malignancy, >20% is considered a high Ki-67 expression, which indicates the high probability of malignancy and a worse prognosis (2), while ≤20% is a low Ki-67 expression.

### MRI acquisition protocol

All patients underwent preoperative DCE-MR examinations using a 3.0T scanner (MAGNETOM Aera, Siemens Healthcare, Erlangen, Germany) with a dedicated 8-channel breast coil. DCE-MR used a spoiled GRE sequence with the following parameters: TR: 4.67 ms, TE: 1.66 ms, flip angle of 10°. The field of view was 360 mm×360 mm, matrix size 384×296, slice thickness 1.2 mm. The scanning process involved initial plain scanning, followed by injection of Gd-DTPA (Magnevist, Bayer Healthcare) 0.2mmol/kg at 2ml/sec through a power injector, which was followed by a 20-mL saline flush. Contrast-enhanced images in six phases were obtained at 60, 120, 180, 240, 300, and 360s after contrast agent injection.

### Image segmentation

**Figure 1:**
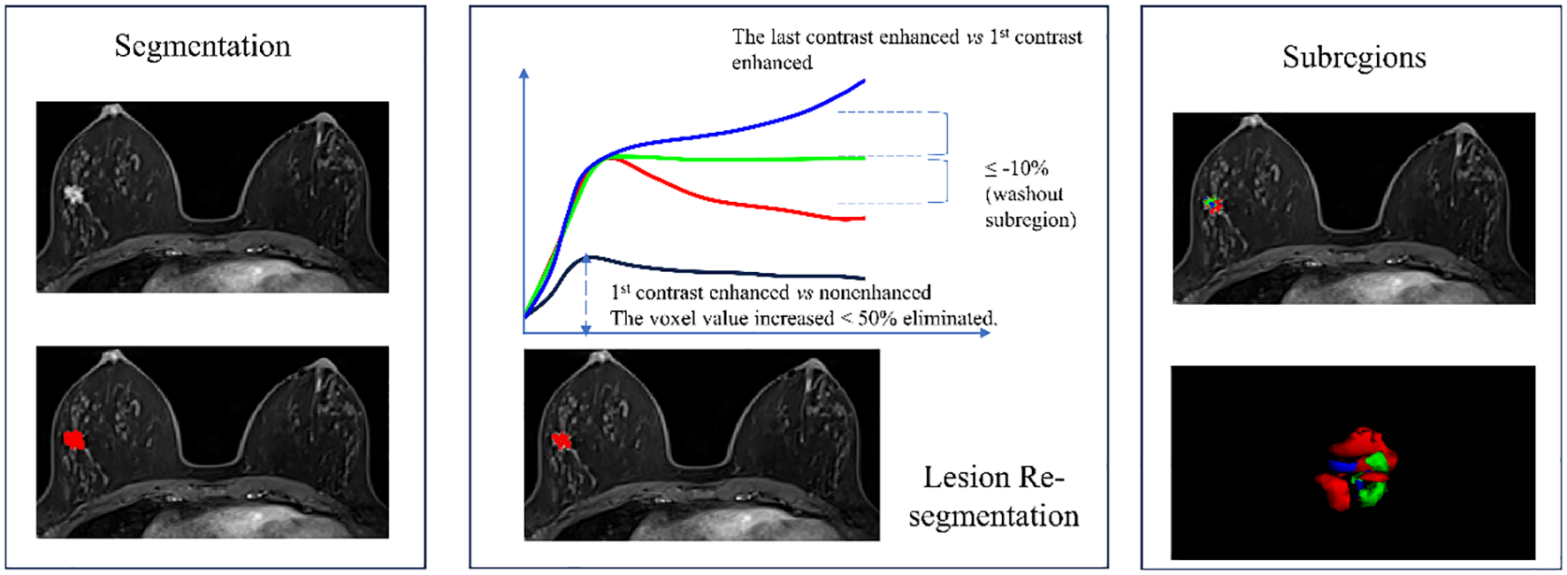
Flowchart of breast cancer lesion segmentation and the 3 subregions re-segmentation. The washout subregion (red) and the plateau subregion (green) were merged as the malignant subregion.

Two experienced breast MRI radiologists conducted breast cancer lesion segmentation. They were blinded to the initial radiological reports and the pathologic outcomes. Radiologist 1 segmented the lesion manually, and Radiologist 2 reviewed the segmentation. If they had different opinions, they would discuss to reach an agreement. The most significantly enhanced phase of the breast DCE image was selected and imported into ITK-SNAP software (version 3.8.0, www.itksnap.org) to identify the tumor outlines (14). Radiologist 1 scanned the entire image axially to locate layers where the lesion existed and then delineated the contours of the lesions layer by layer to obtain a three-dimensional volume of Interest (VOI). VOI was then resegmented to obtain the malignant subregion based on the following principles: firstly, voxels with enhancement ratio >50% were identified by comparing the signal intensity of the first enhanced phase image with the plain phase. Then, comparing the last to the first enhanced phase, voxels with enhancement ratio >10% were classified as the persistent subregion, those with enhancement ratio <-10% as the washout subregion, and the remaining voxels consisted of the plateau subregion (6, 7). The malignant subregion was defined as the combination of the washout subregion and plateau subregion and saved as a separate VOI file for feature extraction. The overall segmentation process is illustrated in **Figure 1**.

### Feature extraction

The VOI corresponding to the malignant subregion and the images of the first enhanced phase were imported into the Pyradiomics software (version 3.0.1, https://pyradiomics.readthedocs.io/en/latest/) to extract the texture features. Pyradiomics, conforming to the Image Biomarker Standardization Initiative (IBSI), is widely used for extracting texture features (15, 16). The feature extraction included two steps: image preprocessing and feature extraction. Image preprocessing involved resampling the image to a voxel size of 1×1×1 mm³, gray discretization using a binWidth=5, and image intensity normalization using the μ ± 3σ method. In order to highlight the value of texture features, additional types of features were also included. Finally, the features encompassed four categories: first-order histogram features, texture features, shape features, and higher-order features extracted from Laplacian of Gaussian (LOG) and wavelet filtered images. In total, 1158 features were extracted, and the comprehensive list of features can be found in the **Supplementary File**.

### Comparison between high and low Ki-67 expression groups

According to the features distribution, an independent sample T-test or Mann-Whitney U test was used to compare the texture features between high and low Ki-67 expression cohorts. A Manhattan plot was used to display the distribution of log-transformed p-values.

### Ki-67 expression predictive model construction and validation

First, a correlation analysis was conducted among the features with significant differences between high and low Ki-67 expression groups. For paired features with a correlation coefficient greater than 0.75, the feature with a higher average correlation with other features was eliminated. Subsequently, the minimum redundancy maximum relevance (mRMR) algorithm was employed for feature selection to identify a feature set that maximally correlates with the Ki-67 expression status while minimizing redundancy among features. This process involved calculating the mutual information between the Ki-67 expression status and each feature, then subtracting the average mutual information of previously selected features from the current feature. The top 5 features were then selected to build a predictive model for Ki-67 expression using a support vector machine algorithm.

The predictive performance of the model was assessed using receiver operating characteristic curve analysis (ROC). The optimal cut-off value for the model was determined based on the Youden index, and the model’s accuracy, sensitivity, specificity, positive predictive value, and negative predictive value were calculated. To verify the reliability of the model, a 10-fold cross-validation was conducted. The average area under the ROC curve (AUC) and diagnostic performance parameters from the cross-validation were also obtained.

### Statistical analyses

All statistical analyses in this study were performed using R statistical software (version 4.2.1, www.r-project.org). Features conforming to a normal distribution were represented by mean ± standard deviation, while others were represented by median and interquartile range. ROC analysis was used to evaluate the predictive performance of texture features and the model, with the AUC as the assessment indicator. A p-value of <0.05 was considered statistically significant.

## Results

### Patient characteristics and pathological results

The study included 119 pathologically confirmed breast cancer patients, with an average age of 54.19 ± 11.05 years. By comparing the clinicopathological information between high and low Ki-67 expression cohorts, the age (High vs. Low: 53.420 ± 9.606 vs. 55.842 ± 13.637, p = 0.264), tumor size (High vs. Low: 2.707 ± 1.390 vs. 2.497 ± 1.749, p = 0.480), Lymph node metastasis (High vs. Low, percentage of Yes: 44.444% vs. 36.842%, p = 0.559) and Lymphovascular invasion (High vs. Low, percentage of Yes: 22.222% vs 21.053%, p = 1.000) shown no significant differences. However, the percentage of WHO III in high Ki-67 expression cohort was significantly higher than the low Ki-67 expression cohort (High vs. Low: 20.988% vs. 2.632%, p=0.006), similarly, high Ki-67 expression cohort occupied higher TNBC percentage (High vs. Low: 33.333% vs. 13.158%, p = 0.036). The detailed information was shown in **Table 1**.

**Table 1:** Comparison of the patients’ clinicopathological information between high and low Ki-67 expression cohorts.

		Ki-67 Status (n = 119)		p-value
		High (n=81)	Low (n=38)	
Age (Year)		53.420 (9.606)	55.842 (13.637)	0.264
Size (mm)		2.707 (1.390)	2.497 (1.749)	0.480
WHO	I	15 (18.519)	15 (39.474)	
	II	49 (60.494)	22 (57.895)	
	III	17 (20.988)	1 (2.632)	0.006
TNBC	No	54 (66.667)	33 (86.842)	
	Yes	27 (33.333)	5 (13.158)	0.036
LNM	No	45 (55.556)	24 (63.158)	
	Yes	36 (44.444)	14 (36.842)	0.559
LVI	No	63 (77.778)	30 (78.947)	
	Yes	18 (22.222)	8 (21.053)	1.000

TNBC, triple negative breast cancer; LNM, lymph node metastasis; LVI, Lymphovascular invasion.

### Differences in texture features between different Ki-67 expression levels

**Figure 2:**
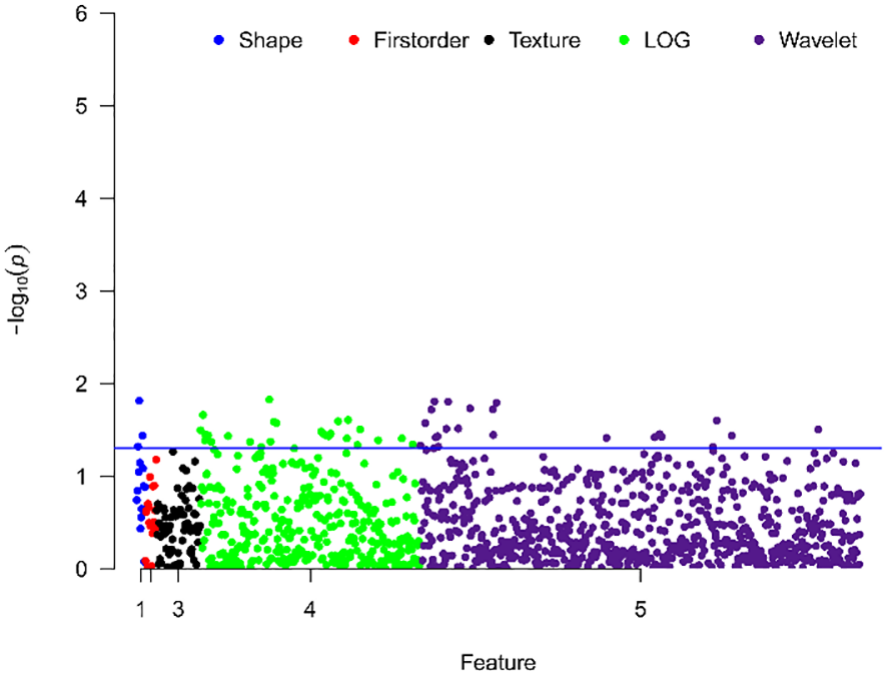
Manhattan plot showing the distribution of p-values for the significance of texture feature differences. The y-axis represents -log10(p), with larger values indicating smaller p-values. Each dot represents a texture feature. The blue line indicates p=0.05, with points above the line representing texture features with significant differences.

Comparison between groups with different Ki-67 expression levels revealed significant differences in 51 texture features, including 48 high-order features (26 from Laplacian of Gaussian transformation, 22 from wavelet transformation), and 3 shape features, no first-order histogram feature and second-order texture features shown significantly different between two cohorts, as shown in **Figure 2**.

### Ki-67 expression predictive model construction and validation

**Figure 3:**
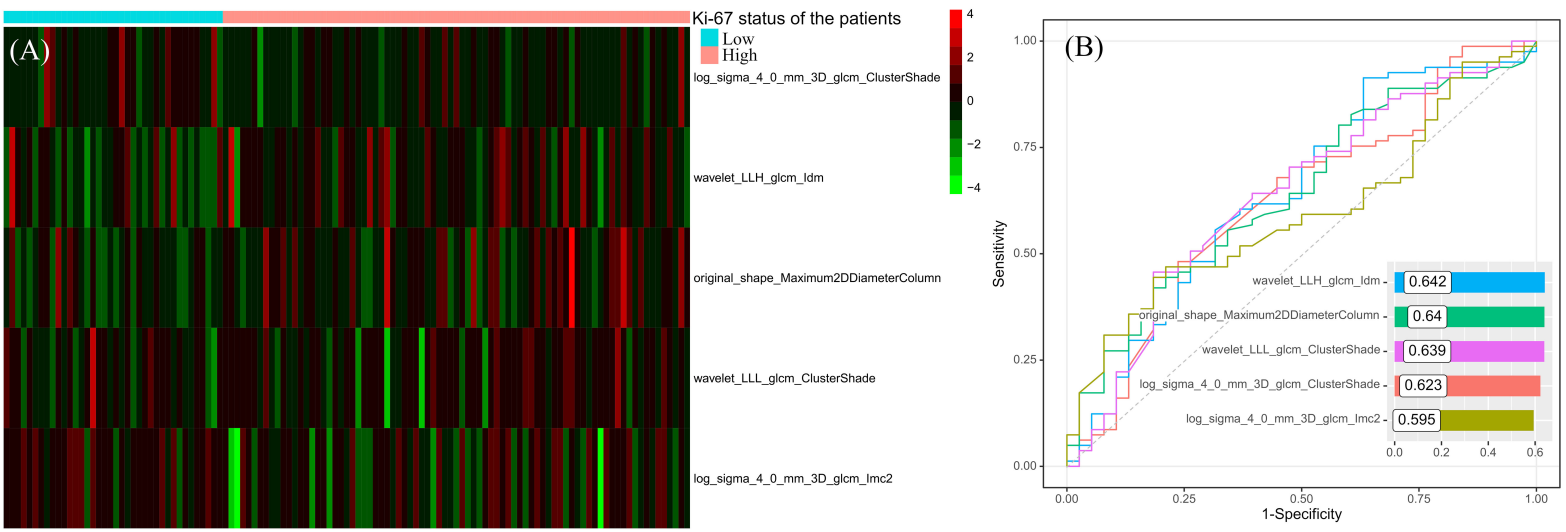
The distribution heatmap of the features used to construct the predictive model in different Ki-67 expression status **(A)** and their results of ROC analyses **(B)**.

After correlation analysis and mRMR, 46 redundant features were excluded, 5 remaining features entered into the SVM classifier to predict the Ki-67 status. The distributions of the 5 features in the high and low Ki-67 expression cohorts are illustrated in **Figure 3A**, highlighting a visually distinct contrast. Through ROC analysis for each feature, the range of AUC values were from 0.595 to 0.642, and wavelet_LLH_glcm_Idm had the highest AUC value (**Figure 3B**).

**Figure 4:**
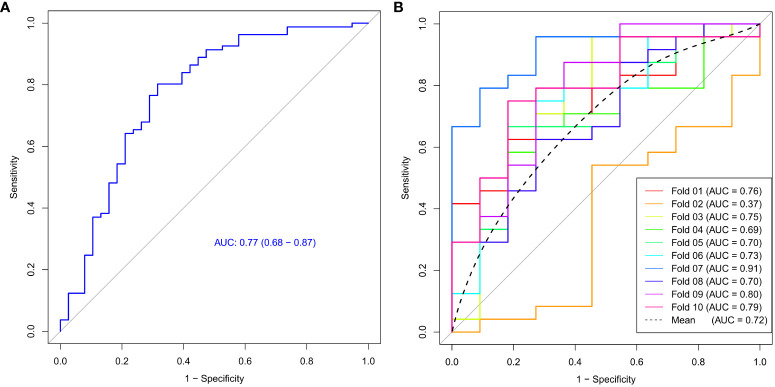
The ROC curve **(A)** and 10-fold cross-validation mean ROC curve **(B)** of the Ki-67 expression model.

The ROC curve of the model was shown in **Figure 4**, with an AUC of 0.77 and a 95% confidence interval of [0.68, 0.87]. Based on the Youden index, the optimal cut-off value for the model was 0.366, with corresponding model accuracy, sensitivity, specificity, positive predictive value, and negative predictive value of 0.765, 0.802, 0.684, 0.844, and 0.619, respectively. To validate the model reliability, the 10-fold cross-validation was conducted and the result was shown in **Figure 4**, with the highest AUC reaching 0.91 and an average AUC of 0.72 ± 0.14.

## Discussion

Previous studies have evaluated breast cancer using the whole lesion in assessing tumor enhancement patterns in DCE-MRI. However, tumor is heterogeneous internally, with different regions showing different enhancement patterns. Following the methodology of Kim et al. (6, 7), in this study, we obtained enhancement curves for individual voxels and classified the tumor into plateau, persistent, and washout subregions. The washout and plateau subregions often correlate with the malignancy of the tumor. Similarly, Ki67, a marker of cell proliferation, is also associated with the malignancy of tumors. Our study was the first to investigate whether the texture features of the malignant subregion (the washout subregion and plateau subregion) could predict Ki-67 expression status.

Various methods were developed to identify the subregions of the tumor, with habitat analysis is the widely used method. The habitat analysis involves obtaining quantitative parameters for each voxel within the tumor, such as Ktrans, Kep, Ve, and Vp from high temporal resolution DCE-MRI, followed by clustering algorithms to cluster the voxels into different subregions. This segmentation method has limitations as the subregions’ physiological information was unclear. Similarly, Fan et al. ever used the subregions in tumor to predict the Ki-67 status, although their subregions considering to the pattern of enhancement, but the subregions also didn’t have clear definition (17). Our approach, based on enhancement curves for subregion segmentation, considered the principles of tumor enhancement. Different enhancement types reflected cells with different levels of malignancy, making the segmentation more clinically meaningful.

Texture analysis has been widely used in noninvasive tumor investigation, and the texture features are able to capture the invisible information correlated with clinical outcome or pathological characteristics (18). In this study, we classified texture features into four categories: shape, histogram, second-order texture, and high-order texture features. However, as shown in the Manhattan plot, there weren’t the first-order histogram features and the second-order texture features significantly differed between high and low Ki-67 expression cohorts. In contrast, a substantial number of high-order texture features derived from Laplacian of Gaussian and wavelet transformed images showed significant differences between high and low Ki-67 expression groups. This suggested that the malignant subregion, also related to tumor malignancy, was influenced by Ki-67 expression levels. Lower-order features, which are not visually discernible, do not represent molecular-level information. In contrast, high-order features captured more detailed information within lesions and were more sensitive to molecular-level differences (12).

We constructed a model for predicting Ki-67 expression levels using support vector machine algorithm and evaluated its efficacy with 10-fold cross-validation. The model’s AUC reached 0.77, with an average AUC of 0.72 for cross-validation, suggesting that the machine learning model based on the features derived from the malignant subregion could accurately and reliably predict Ki-67 expression levels, offering significant diagnostic value for breast cancer. Simultaneously, we noted a low AUC value for fold 2, possibly stemming from the varying distribution of features within fold 2 compared to the rest of the data. Therefore, conducting cross-validation is essential to mitigate such occasional discrepancies and validate the robustness of the predictive model, with the averaged AUC holding more valuable.

The Ki-67 predictive model included 1 shape feature and 4 high-order texture features. The shape feature was original_shape_Maximum2DDiameterColumn, which was defined as the largest pairwise Euclidean distance between tumor surface mesh vertices in coronal plane. In high Ki-67 expression patients, original_shape_Maximum2DDiameterColumn had the higher value, which suggested high Ki-67 expression leaded to the malignant subregion having longer diameter in coronal plane. In 4 high-order texture features, wavelet_LLH_glcm_Idm is a measure of local homogeneity of the image, which increased in high Ki-67 expression cohort. This suggested that although high Ki-67 expression indicates a higher malignancy level and higher malignancy is typically associated with increased tumor heterogeneity, the malignant subregion of the tumor tended to show consistent enhancement patterns when Ki-67 expression was high. The wavelet_LLL_glcm_ClusterShade and log_sigma_4_0_mm_3D_glcm_ClusterShade are both the measures of the skewness and uniformity of the gray level co-occurrence matrix (GLCM), which suggested that in high Ki-67 expression cohorts, their malignant regions’ GLCMs were low skewness. The log_sigma_4_0_mm_3D_glcm_Imc2 quantified the complexity of the texture, which was lower in high Ki-67 expression cohorts, in other words, the microscopic information in the malignant subregion tends to be more consistent.

However, this study has several limitations. Firstly, there was no independent external validation to assess the performance of the predictive model and their reproducibility. However, we utilized cross-validation to confirm the reliability of the model. Secondly, the value of the persistent subregion’s texture features in predicting the Ki-67 expression status was not investigated and compared with the other subregions. In future research, we would include the other regions, such as peritumor region, to identify the best region to predict Ki-67 expression status.

## Data availability statement

The raw data supporting the conclusions of this article will be made available by the authors, without undue reservation.

## Ethics statement

The studies involving humans were approved by Research Ethics Committees of Changzhou Cancer Hospital. The studies were conducted in accordance with the local legislation and institutional requirements. The ethics committee/institutional review board waived the requirement of written informed consent for participation from the participants or the participants’ legal guardians/next of kin because Our study is a retrospective study. We collected patients who had breast cancer examination in our hospital before, and did not bring any privacy leakage to patients, which did not affect the normal diagnosis and treatment of patients.

## Author contributions

CH: Conceptualization, Data curation, Formal analysis, Investigation, Methodology, Project administration, Resources, Validation, Visualization, Writing – original draft, Writing – review & editing. CW: Data curation, Supervision, Writing – original draft, Writing – review & editing. RH: Conceptualization, Formal analysis, Methodology, Software, Validation, Writing – original draft, Writing – review & editing. PT: Conceptualization, Data curation, Investigation, Resources, Writing – original draft, Writing – review & editing. ZJ: Conceptualization, Funding acquisition, Investigation, Project administration, Supervision, Writing – original draft, Writing – review & editing.
